# Impacts of an Invasive Snail (*Tarebia granifera*) on Nutrient Cycling in Tropical Streams: The Role of Riparian Deforestation in Trinidad, West Indies

**DOI:** 10.1371/journal.pone.0038806

**Published:** 2012-06-25

**Authors:** Jennifer M. Moslemi, Sunny B. Snider, Keeley MacNeill, James F. Gilliam, Alexander S. Flecker

**Affiliations:** 1 Department of Ecology and Evolutionary Biology, Cornell University, Ithaca, New York, United States of America; 2 Department of Biology, North Carolina State University, Raleigh, North Carolina, United States of America; 3 School of Natural Resources, University of Nebraska, Lincoln, Nebraska, United States of America; University of Western Australia, Australia

## Abstract

Non-native species and habitat degradation are two major catalysts of environmental change and often occur simultaneously. In freshwater systems, degradation of adjacent terrestrial vegetation may facilitate introduced species by altering resource availability. Here we examine how the presence of intact riparian cover influences the impact of an invasive herbivorous snail, *Tarebia granifera*, on nitrogen (N) cycling in aquatic systems on the island of Trinidad. We quantified snail biomass, growth, and N excretion in locations where riparian vegetation was present or removed to determine how snail demographics and excretion were related to the condition of the riparian zone. In three Neotropical streams, we measured snail biomass and N excretion in open and closed canopy habitats to generate estimates of mass- and area-specific N excretion rates. Snail biomass was 2 to 8 times greater and areal N excretion rates ranged from 3 to 9 times greater in open canopy habitats. Snails foraging in open canopy habitat also had access to more abundant food resources and exhibited greater growth and mass-specific N excretion rates. Estimates of ecosystem N demand indicated that snail N excretion in fully closed, partially closed, and open canopy habitats supplied 2%, 11%, and 16% of integrated ecosystem N demand, respectively. We conclude that human-mediated riparian canopy loss can generate hotspots of snail biomass, growth, and N excretion along tropical stream networks, altering the impacts of an invasive snail on the biogeochemical cycling of N.

## Introduction

Species introductions and habitat degradation are two major components of environmental change [1]. Numerous studies have documented the impacts of invasive animals at various levels of biological organization [2], [3], [4]; however, consequences of species invasions on nutrient fluxes have been relatively understudied despite the potential significance for fundamental ecosystem processes. Animals can directly influence ecosystem-scale nutrient fluxes through consumption and excretion of biologically important compounds [5]. Non-native species may alter nutrient fluxes by: (1) altering the biomass of consumers that recycle nutrients at given mass-specific rates [6], or (2) altering assemblages of organisms and their corresponding mass- and species-specific nutrient excretion ratios [7], [8].

Previous studies have shown that individual species can play important roles in nutrient cycling, even in tropical aquatic systems with diverse communities [9], [10], [11]. This lends support to the argument that the addition of new species can have potentially large consequences on tropical biogeochemical cycles (e.g. *Pomacea canaliculata*) [12]. Also, nutrient cycles and controls on primary production may differ between tropical and temperate systems, with nitrogen more limiting in tropical freshwaters and phosphorus more limiting in temperate waters (though the strength of this correlation is in question) [13], [14]. Differences in nutrient limitation of producers across temperate and tropical zones suggests that biogeochemical consequences of invasive species studied in temperate zones cannot necessarily be extrapolated to analogous invasions in tropical zones.

Contemporary species invasions occur in the context of rapidly changing landscapes. Habitat degradation caused by land conversion is a prevalent agent of environmental change that can occur simultaneously with invasive animal dispersal and establishment, often with facilitating or unknown effects on invaders [15], [16]. Yet consequences of habitat loss and animal invasions are most often studied independently of one another, without consideration of potential interactions [17]. Lack of understanding of interactive effects hinders efforts to predict and manage invasions at multiple scales. Identification of land use changes that unintentionally facilitate invaders, yet can be reasonably curtailed or reversed, is useful for developing feasible mitigation strategies. Degradation of riparian forests adjacent to water bodies may be of heightened importance for aquatic ecosystem dynamics because of the potential for disruptions to resource flux between terrestrial and aquatic ecosystems [18], [19]. Aquatic non-native species can also decouple nutrient exchange between terrestrial and aquatic ecosystems [4], although it remains unclear whether the combination of invasive species and riparian degradation can have interactive properties that exceed the additive impacts of individual threats.

Here we studied how riparian canopy cover alters the impacts of an introduced herbivore/detritivore, the quilted melania snail (*Tarebia granifera*), on nitrogen (N) cycling in tropical streams. Nitrogen is an important and potentially limiting or co-limiting nutrient for primary production in tropical streams [13], [14], [20] and impacts on N cycles may therefore have consequences on aquatic food webs and the services they provide for tropical regions, such as food production for local people. We measured *T. granifera* biomass, growth rates, and N excretion in locations where riparian vegetation was present or removed to determine how these variables were related to the condition of the riparian zone. Phosphorus (P) excretion by snails can also be significant in aquatic systems [12], but P excretion by *T. granifera* in our study system was not detectable from background levels of dissolved P in streamwater (J. Moslemi, *unpublished data*). We also compared total snail N excretion to ecosystem N demand to estimate the degree to which *T. granifera* influences N cycling in tropical streams, and how that influence may be mediated by riparian canopy cover. Lack of riparian vegetation could be hypothesized to have opposing impacts on food resource availability and, subsequently, on snail biomass, growth, and excretion rates - it may augment sunlight availability and lead to increases in algal productivity and biomass, but also inhibit subsidies of allochthonous food resources from terrestrial ecosystems. We determined the overall quantity and quality of food resources available to invasive snails in habitats where riparian vegetation was present and removed, and discuss the potential implications for snail demographics, metabolism, and impact on ecosystem-scale N cycling.

## Methods

### Study Species

**Figure 1:**
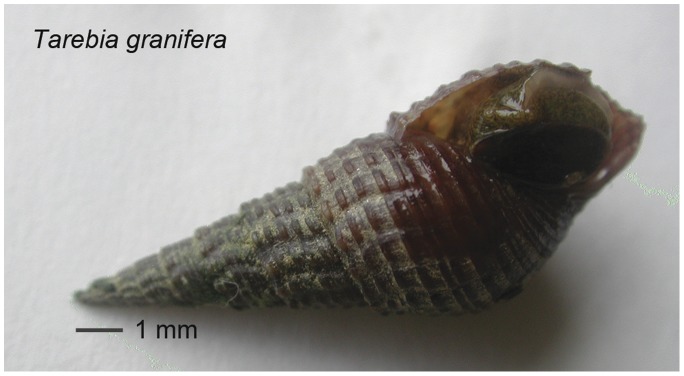
*Tarebia granifera.* The quilted melania snail has invaded freshwater habitats throughout much of the Neotropics. Photo credit: S. B. Snider.

Introduction of *Tarebia granifera* (Gastropoda: Thiaridae) to the West Indies occurred via both accidental and intentional pathways (Fig. 1). Initial introduction of this parthenogenetic snail to the Neotropics from Asia is presumed to have been a consequence of the aquarium trade, though intentional introductions have occurred in an attempt to reduce schistosomaisis outbreaks [21]. *Tarebia granifera* is now found worldwide throughout the topics and subtropics, though the ecological impacts of invasion remain largely unknown.

### Study Site

We focused on three streams for this study–Ramdeen Stream (RAM), Aripo River (ARI), and Yarra River (YAR)–from separate drainage basins in the Northern Range Mountains on the island of Trinidad, West Indies (10°41′ N, 61°17′ W). Nutrient diffusing substrates [22] placed in RAM in 2007 indicated N and phosphorus co-limitation of primary production (Supporting information S1, Fig. S1).

### Field and Laboratory Methods

Percent riparian canopy cover over streams was determined using a densiometer (Type A convex, CSP Outdoors, Shreveport, LA, USA). “Open canopy” habitat was designated as 25% canopy cover or less, “closed canopy” habitat was 75% canopy cover or greater, and remaining values (26–74% canopy cover) were categorized as “partially closed canopy”. Quantity and quality of food resources for invasive snails were characterized in open and closed canopy habitats by measuring biomass of organic matter (chlorophyll *a* and ash-free dry mass) and carbon:N composition of epilithon, respectively. Epilithon was collected by scrubbing the entire surface area of rocks on the stream bottom with a plastic bristle brush, generating a slurry of organic and inorganic matter (3 to 5 rocks scrubbed per slurry, 5 slurries per site). A subsample of known volume was removed from the slurry using a pipette and filtered (Pall-Gelman, Type A/E 25 mm or 47 mm) for analysis of chlorophyll *a* (as an indicator of algal biomass) and ash-free dry mass (AFDM; as an indicator of organic material biomass). Upon filtration, chlorophyll *a* samples were extracted in 90% buffered ethanol for 24 hours and analyzed using standard fluorometric techniques [23] (Turner Designs Aquafluor, Sunnyvale, California, USA). AFDM samples were processed following methods in [24]. To generate areal estimates of algal biomass and AFDM, we traced rocks used for each slurry and calculated areas of tracings using ImageJ software (National Institutes of Health, Bethesda, Maryland, USA). The remaining slurry volume was settled in refrigerated conditions over a 24 h period after which water was decanted and the remaining concentrated sample dried in a drying oven (temperature  = 55°C) for subsequent elemental composition analysis at Cornell University. Once transported to the laboratory, samples were homogenized and weighed on a microbalance (Mettler Toledo MX5, Columbus, OH, USA) to the nearest ug. Carbon (C) and N content of samples were analyzed using an elemental analyzer (Elementar Vario EL III, Frankfurt, Germany).

*Tarebia granifera* densities were estimated for each habitat type from randomly assigned transects (RAM) or quadrats (ARI and YAR) using a hand net (2 mm mesh size; 5 total sites for each habitat type). Cross-stream transects of a constant width (0.15 m) from the edge to the midpoint of stream wetted width were used in RAM (*n* = 16). Quadrats (0.33 m^2^) in ARI (*n* = 16) and YAR (*n* = 12) were divided evenly between random locations across the width of streams to account for edge effects. Total length of individual snails was measured to the nearest 0.1 mm using digital calipers. Snails smaller than 2 mm were not included in the study. Snail biomass was measured as mg of ash-free dry mass (AFDM) and calculated using a length-mass regression (log AFDM  = 2.35*L* –1.83, where *L* is log of shell length in mm; *n* = 34, *r^2^* = 0.98) or an aperture-mass regression if shell tips were eroded or broken (log AFDM  = 2.45*A* –0.08, where *A* is log of shell aperture width in mm; *n* = 25, *r^2^* = 0.96).

We measured *T. granifera* N excretion rates using methods modified from [6]. In 2008, individual snails were placed into 20 ml, clear plastic vials filled with filtered stream water. Snails were collected during daylight hours and immediately incubated in vials for 1 h in the field. After the incubation, water samples were filtered (Pall-Gelman A/E) and analyzed for NH_4_ concentration using fluorometric methods described in [25]. We focused on NH_4_ because it is the dominant N compound excreted by aquatic snails and is readily available for uptake by primary producers and microorganisms. Sampled snails represented the size distribution of *T. granifera* found in each stream (RAM: *n* = 124, ARI: *n* = 40, YAR: *n* = 40). We controlled for background NH_4_ concentration in stream water and any vial effects by subtracting the background NH_4_ concentration in vials incubated without snails (RAM: *n* = 16, ARI: *n* = 4, YAR: *n* = 4). Some fraction of calculated NH_4_-N excretion rates may have been due to leaching from egested fecal matter. Egestion was not quantified but fecal production was low over the course of incubations. We estimated areal N excretion by multiplying mass-specific excretion rates by average snail density per unit streambed area for each snail size class, then aggregating across all size classes.

To determine if *T. granifera* growth rates differed between canopy types, we measured growth rates of snails using a reciprocal transplant design in RAM. We chose an open canopy and closed canopy site separated by 30 m. This was considered sufficient distance between independent open and closed canopy sites as previous work determined that average longitudinal movement rates for *T. granifera* individuals in RAM were 3.7±0.15 m/week (*n* = 220 individuals) [26]. We collected 50 snails in each open and closed canopy site; of those, half were placed in the same site (as a control) and the remaining half were transplanted to the reciprocal site. Snails were collected over a broad area within each canopy site to ensure that the sample was representative of the source population. Snails were placed in groups of five into separate flow-through containers along with substrate collected from the incubation site to alleviate potential starvation effects. Length of each snail was measured before the experiment and after 10 days of incubation. Growth rates were calculated as (ln *M_t_* – ln *M_0_*)/*t* where *M_t_* was the mass of an individual snail after incubation and *M_0_* was the mass of the same snail before incubation [27]. We used response ratios to estimate effect sizes and 95% confidence intervals of biomass-specific snail growth rates: ln(*X_t_*/*X_c_*), where *X_t_* is the mean growth rate for transplanted snails and *X_c_* is the mean growth rate for control snails [28].

To put *T. granifera* NH_4_-N excretion into the context of ecosystem-level NH_4_-N demand, we measured area-specific NH_4_-N uptake rate in RAM in February 2008 and 2010 [29]. Due to lack of open canopy sites in 2010 caused by regrowth of riparian vegetation, comparisons of snail N excretion and ecosystem demand for that year are between partially and fully closed canopy habitats as opposed to open and closed canopy habitat. NH_4_-N demand was measured using a short-term solute addition [22] in which known concentrations of NH_4_ (as NH_4_Cl) and a conservative tracer (NaCl) were simultaneously released at a constant rate via a peristaltic pump. The decline in concentration of NH_4_ was measured after correction for background concentration and dilution (using the decline in conservative tracer as an estimate). Solutes were released until conductivity reached plateau at the downstream end of the study reach (∼1 h). Sampling stations were set up every 10 m along a 100 m study reach; NH_4_ samples and conductivity measurements were taken at all stations before solutes were added (to establish background concentrations) and once conductivity had reached plateau (*n* = 3 per station). Conductivity was measured using a YSI 85 meter (Yellow Springs, OH, USA). Water samples were analyzed fluorometrically within 4 hours of sampling. Mass of NH_4_ added to the stream was calibrated to be detectable using fluorometric techniques but not in sufficient quantities to alter pathways of uptake over the time period of the addition (∼2 h). We used data from the solute addition to calculate NH_4_-N uptake length (the length an NH_4_^+^ ion travels downstream before biotic uptake), uptake velocity (the velocity at which biotic uptake removes N from the water column), and areal uptake rate (Supporting information S2). Short-term nutrient releases can underestimate demand of algae and microbes [30]. To minimize this potential error we kept N addition low (2–5 times ambient NH4-N concentrations) while still allowing for detection at downstream transects.

Tests for significance of riparian canopy effect across streams were conducted using a mixed effects ANOVA model using canopy cover as a fixed effect and stream as a random effect. Tests of canopy effects within individual streams were conducted using Student’s *t* (SAS Institute, 2009). Data on *T. granifera* biomass density and N excretion rates were log_10_-transformed to satisfy ANOVA assumptions of normality and equal variance.

## Results

**Figure 2:**
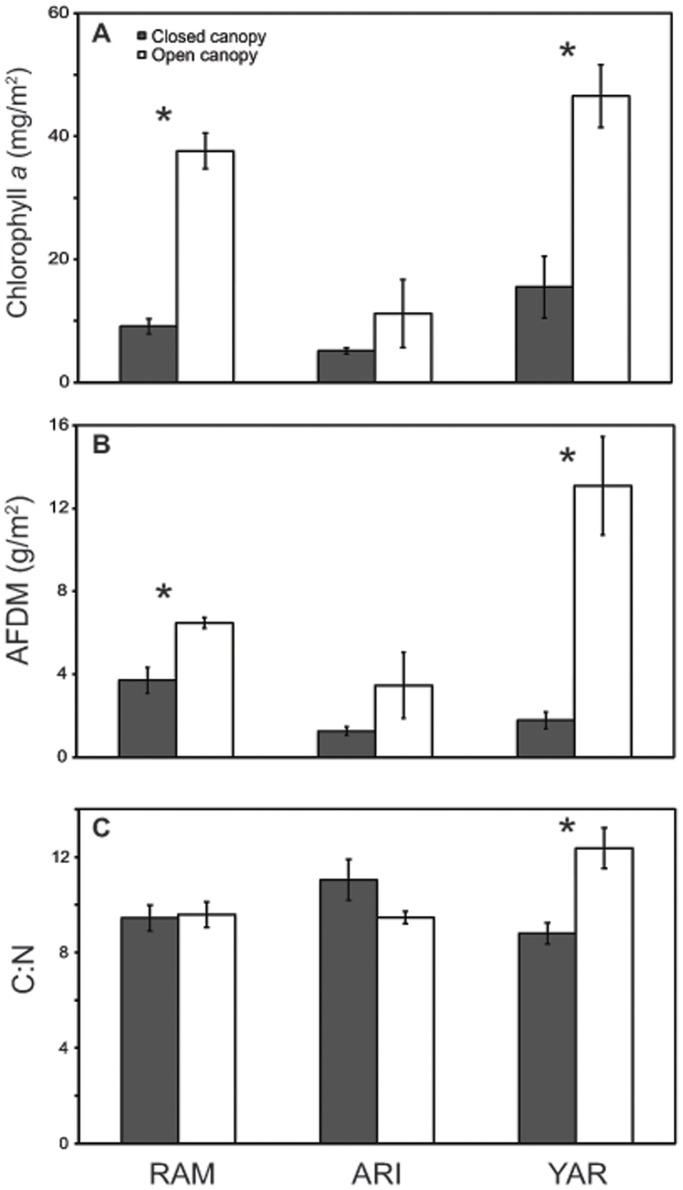
Quantity and quality of snail food resources by canopy cover state. Mean (±1 SE) chlorophyll *a* (A), ash-free dry mass (AFDM) (B), and molar C:N ratios of epilithon (C). RAM  =  Ramdeen Stream, ARI  =  Aripo River, YAR  =  Yarra River. Gray and white bars represent data collected in closed and open canopy sites, respectively (“closed” is ≥75% and “open” is ≤25% canopy cover). Asterisks above bars represent significant differences (*p*<0.05) among canopy types within streams.

Food quantity, measured as algal biomass (chlorophyll *a*) and organic matter (AFDM), was significantly greater in open canopy habitats (chlorophyll *a*: *F*_1,28_ = 33.52, *p*<0.001; AFDM: *F*_1,28_ = 18.84, *p*<0.001; Fig. 2). Within-stream comparisons indicate that food quantity as measured by epilithon molar C:N ratios was greater in RAM (chlorophyll *a*: *t*_1,8_ = 9.05, *p*<0.001; AFDM: *t*_1,8_ = −4.14, *p*<0.003) and YAR (chlorophyll *a*: *t*_1,8_ = 4.89, *p*<0.001; AFDM: *t*_1,8_ = −4.72, *p*<0.002) but not ARI. Epilithon molar C:N ratios did not differ among canopy types in RAM and ARI, but were greater where canopy was removed in YAR (*t*_1,8_ = −3.73, *p*<0.01; Fig. 2). Elemental analysis of *T. granifera* body C:N and C:P content (shell removed) in RAM and ARI showed no difference among canopy types (Supporting information S3, Table S1).

**Figure 3:**
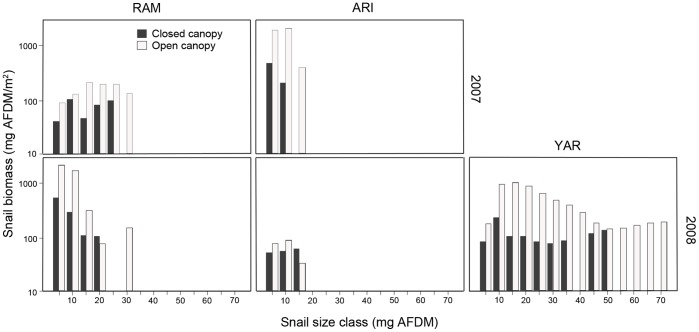
Mean *T. granifera* biomass by size class. Top row of panels are 2007 data and bottom row are 2008 data. Size classes were based on 5 mg AFDM increments. RAM  =  Ramdeen Stream, ARI  =  Aripo River, YAR  =  Yarra River. Gray and white bars represent data collected in closed and open canopy sites, respectively. Canopy type had a significant impact on *T. granifera* areal biomass (*F* = 22.06, *p*<0.0001). Note log scale. No data were collected in YAR in 2007.

Average snail density ranged from 23 individuals/m^2^ (ARI closed canopy, 2008) to 1424 individuals/m^2^ (RAM partial canopy, 2010). Canopy cover negatively influenced *T. granifera* biomass across the three study streams (*F*_1,38_ = 22.06, *p*<0.001), and the range of snail size distributions varied among streams (Fig. 3). Within-stream comparisons indicated that snail biomass was greater in open canopy habitats in all streams, ranging from 2 to 8 times the densities found in closed canopy habitats (RAM: *t*_1,10_ = −4.84, *p*<0.001; ARI: *t*_1,14_ = −2.16, *p* = 0.047; YAR: *t*_1,10_ = −2.67, *p* = 0.028). In 2010, no open canopy sites remained in the RAM study site due to riparian vegetation growth; however, snail densities were higher in habitats that were only partially closed compared to those with a fully closed canopy (Mean individuals/m^2^±1SD: partially closed canopy  = 1424±357, fully closed canopy  = 578±218; *t*_1,10_ = −2.02, *p* = 0.071).

**Figure 4:**
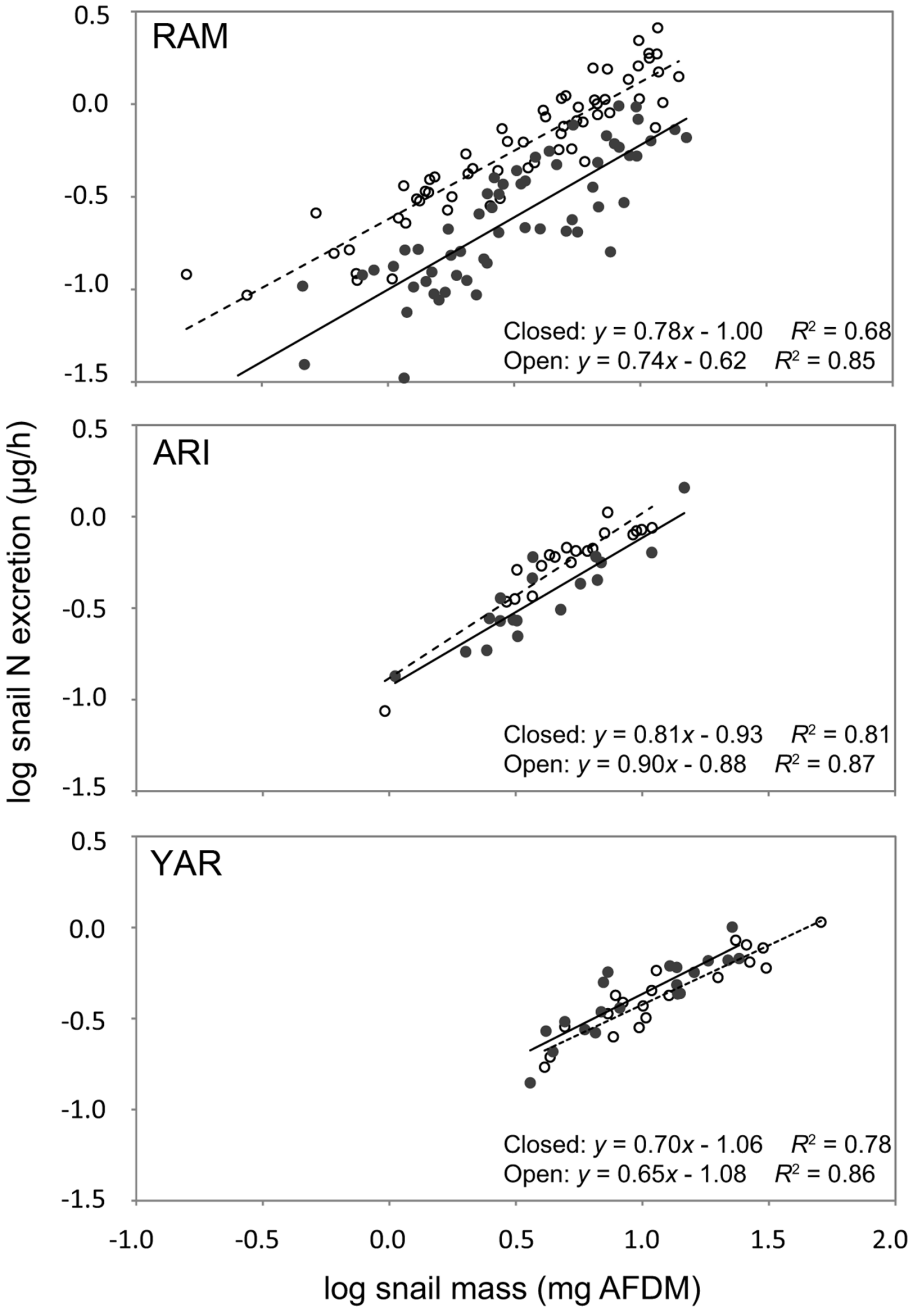
Influence of canopy state on nitrogen excretion by *T. granifera* in three streams. Panels from top represent Ramdeen Stream (RAM), Aripo River (ARI), and Yarra River (YAR). Open and closed circles represent individual snails collected in open and closed canopy habitats, respectively. Solid lines represent trends in closed canopy habitats, and broken lines represent trends in closed canopy habitats. All data were log-transformed.

Nitrogen excretion rates of individual snails ranged from 0.33 to 2.58 ug N/h. Mass-specific excretion of individual snails decreased with increasing snail size and ranged from 0.02 to 0.76 ug N/mg AFDM/h. Snail N excretion rates showed allometric size scaling and differed significantly among canopy types (Fig. 4; *F*_1,141_ = 86.9, *p*<0.001). Within-stream comparisons indicated that snails in open canopy habitat exhibited greater excretion rates than closed canopy snails in RAM and ARI (RAM: *t*_1,60_ = 5.73, *p*<0.001; ARI: *t*_1,37_ = 2.07, *p* = 0.048), but not YAR.

**Figure 5:**
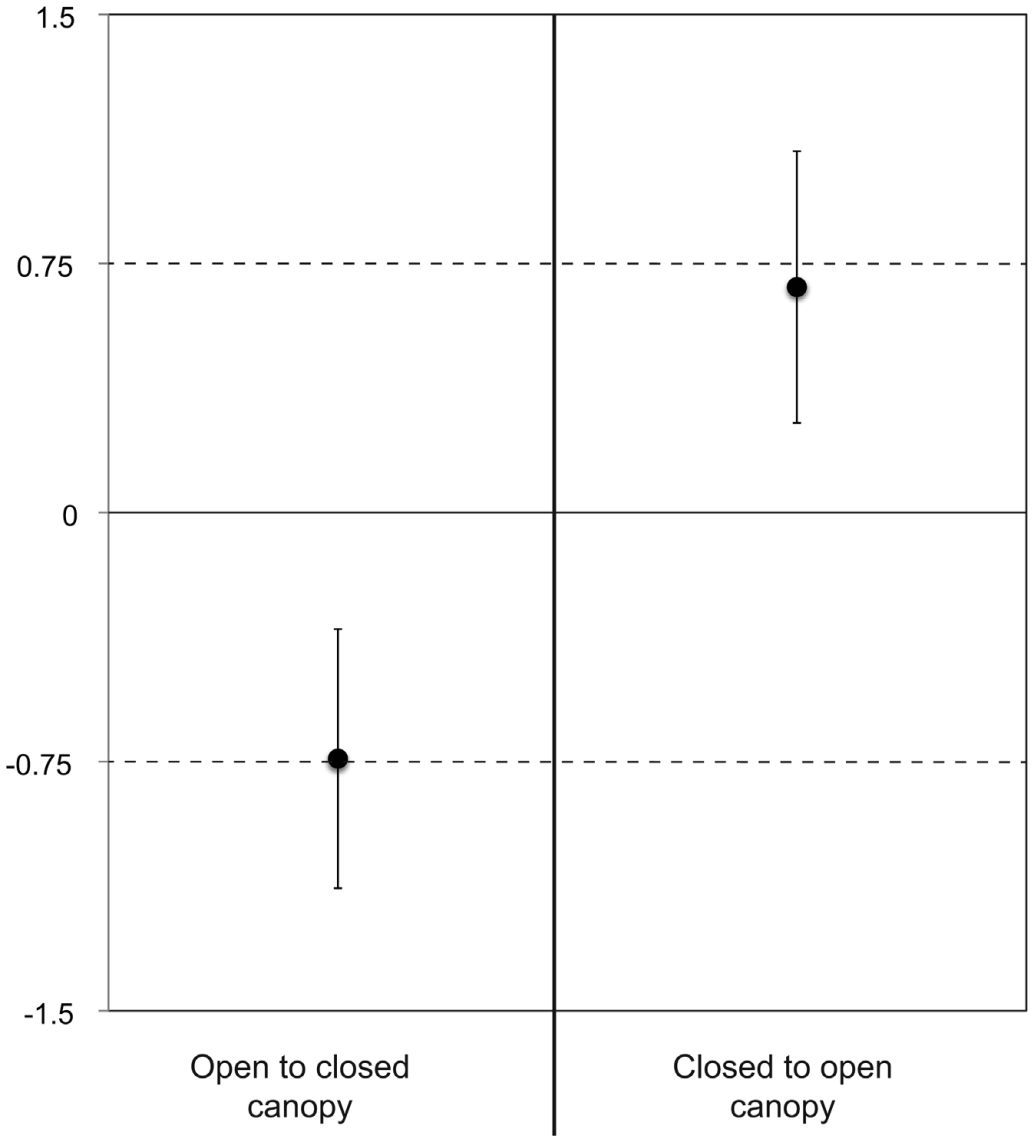
Canopy state and snail growth rates. Effect sizes of canopy state on snail growth rates as measured in a reciprocal transplant experiment. Error bars represent 95% confidence intervals and effect sizes were measured as response ratios (Hedges et al. 1997). “Open to closed canopy” represents snails collected from open canopy habitat and moved to closed canopy habitat where growth rates were measured after 10 days, and “closed to open canopy” is vice versa.

Mass-specific growth rates of *T. granifera* measured in a reciprocal transplant experiment were greater in open canopy habitat regardless of treatment (control or transplanted; *F*_1,89_ = 39.82, *p*<0.001). Response ratios were similar for both transplants (0.74 for open to closed canopy, 0.68 for closed to open canopy, Fig. 5). Confidence intervals (95%) did not cross zero, indicating a strong impact of canopy type on snail growth rates. Small snails grew faster than large snails across all treatments (*F*_1,89_ = 26.77, *p*<0.0001) and initial snail size (before incubation) explained 23% of the variation in growth rates for the entire data set.

**Figure 6:**
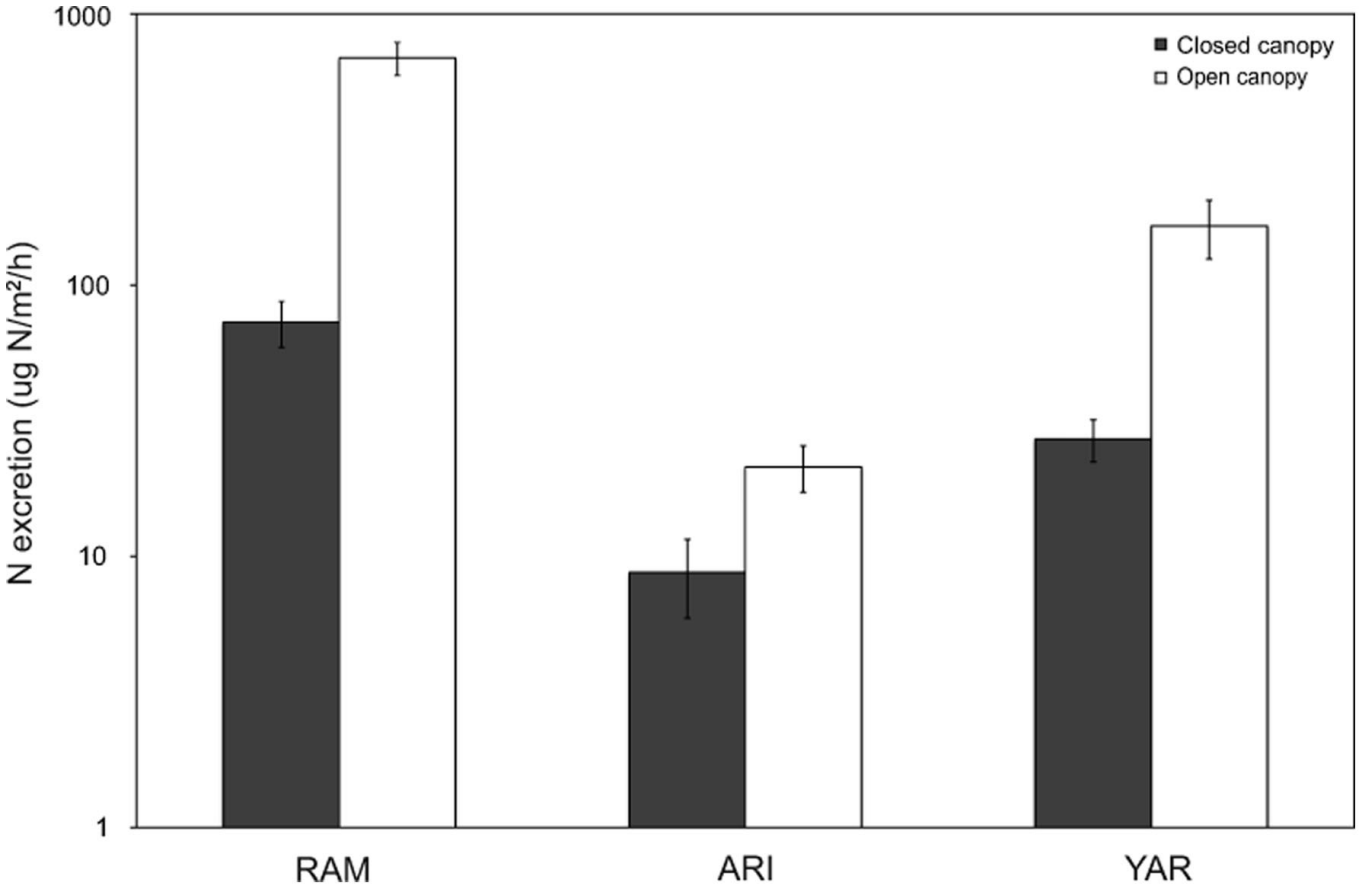
Influence of canopy state on areal snail excretion rates in three streams. Mean (±1 SE) areal N excretion by *T. granifera* in 2008. RAM  =  Ramdeen Stream, ARI  =  Aripo River, YAR  =  Yarra River. Closed and open bars represent data collected in closed and open canopy sites, respectively. Canopy effect was significant across all streams (*p*<0.0001).

Areal excretion rates of *T. granifera* in 2008 (RAM, ARI, and YAR) ranged from 0 to 900 ug N/m^2^/h and were 3 to 9 times greater in open relative to closed canopy habitats. Differences in areal N excretion across canopy types for all three streams were significant (Fig. 6, *F*_1,38_ = 19.4, *p*<0.0001); within-stream analyses indicated areal excretion rates were greater in open canopy habitat across all streams (RAM: *t*_1,14_ = −6.36, *p*<0.001; ARI: *t*_1,10_ = −2.52, *p = *0.025; YAR: *t*_1,10_ = −3.41, *p*<0.01).

Estimates of NH_4_-N uptake rate in RAM in 2008 and 2010 from NH_4_ release data showed a significant decline in background-corrected NH_4_-N with distance downstream from the nutrient addition site (*R^2^* = 0.75 and 0.99, respectively; Fig. S2). Using these data we calculated NH_4_-N uptake length (2008∶75.8 m; 2010∶25.1 m), uptake velocity (2008∶5.7 mm/min; 2010∶10.2 mm/min), and area-specific uptake (2008∶4.19 mgN/m^2^/h; 2010∶7.18 mgN/m^2^/h). Mean areal NH_4_-N excretion rates in fully closed (2008 and 2010), partially closed (2010 only), and open canopy (2008 only) sites supplied 2%, 11%, and 16% of integrated areal NH_4_-N demand, respectively. In 2010, partially closed canopy sites were used instead of open canopy sites for estimates of areal excretion rates and ecosystem N demand, as riparian vegetation regrowth precluded the presence of open canopy sites.

## Discussion

Our results suggest that invasive snails were heterogeneously distributed along streams due to differences in light availability. Human-mediated removal of riparian vegetation can therefore create hotspots of invasive snail excretion and subsequent impacts on aquatic N cycling. The greater influence of *T.granifera* in open canopy sites was linked to increased snail density and, to a lesser extent, increased mass-specific excretion rates relative to shaded sites. Studies have shown that invasive mollusks can alter nutrient cycles in aquatic systems in temperate [6], [7] and tropical [12] zones; here, we have shown that the potential of a single invasive species to impact N cycling in tropical streams is modified by the removal of vegetation in adjacent riparian areas.

Differences in invasive snail impact among sites with different canopy cover were likely driven by variation in food resources. In two out of the three study streams, estimates of algal and organic matter standing crop were significantly greater in open canopy sites where direct sunlight reaches the streambed and primary producers may be released from growth constraints posed by light limitation (Fig. 2). In the Aripo River, estimates of food quantity did not differ among canopy types, perhaps due to unusually high rainfall in the 2008 dry season. The Aripo River is the largest of the three study streams, and intense scouring in this river relative to the other two study streams could have eroded spatial structure in algal biomass. Our results are consistent with studies linking invertebrate production and food resource quantity [31], [32], [33]. In a landscape with heterogeneously deforested riparian zones, increased food availability in open canopy sites may act as spatial resource subsidies, driving increases in snail densities that “spill over” to less desirable closed canopy sites [34]. Due to the subsidizing effect of riparian deforestation on aquatic primary producers, it seems possible that continued removal of riparian canopy cover could facilitate other herbivorous invasive species, though additional research is necessary to confirm this hypothesis.

We found that increased light availability was more often associated with quantity as opposed to quality of snail food resources. Yarra River was the only one of three study streams in which food quality was significantly greater in closed canopy habitat, and interestingly, the only stream for which the effect of canopy type on mass-specific excretion rates was not significant. In this stream, increased algal quality in closed canopy sites may have diminished the benefits of open canopy habitat where food was more plentiful but of poorer quality. These results partially support the light-nutrient hypothesis, which states that increased light intensities should be associated with decreased nutrient content in algal communities [35]. However, as corroborated by our results, support for the light-nutrient hypothesis has not been consistent in benthic systems and several studies have not found a negative effect of light availability on epilithon nutrient content [36], [37], [38], [39]. This may be due to interacting factors such as the availability of dissolved nutrients and the intensity of nutrient limitation of epilithon. Epilithon communities less limited by nutrient availability may be less likely to exhibit decreased nutrient content as a result of high photosynthesis rates [38].

Invasion by *T. granifera* likely has altered a suite of ecosystem properties within our study area. Lack of preinvasion datasets or comparable reference sites uninvaded by snails prohibited direct analysis of ecosystem-level effects of invasion. However, results from this study have shown that primary production in Trinidadian lotic systems can be co-limited by availability of N and phosphorus. Changes in absolute and relative availability of these nutrients mediated by invasive snail excretion may influence the identity of the nutrient that limits primary productivity, as well as the growth and community composition of primary producers [5]. These impacts on basal resources may alter food web dynamics, with unknown consequences on aquatic consumers. In addition, since few predators have been observed to consume *T.* *granifera* in Trinidadian streams despite dense and conspicuous aggregations (S.B. Snider, *unpublished data*), these primary consumers may act as a trophic dead end for aquatic communities, diverting energy fixed by primary producers away from higher trophic levels [40].

Snail size distributions influenced aggregate N excretion among study streams. Small-bodied organisms generally exhibit greater mass-specific excretion rates due to higher metabolism relative to large-bodied organisms [41]. Differences in snail size distributions among our study streams influenced areal excretion rates, as smaller snails excreted relatively more per unit mass than larger snails. In Ramdeen Stream, snail size distribution was characterized by dense aggregations of smaller individuals, whereas the Yarra population contained smaller aggregations of larger snails (Fig. 3). The difference in size structure led to increased areal excretion rates in Ramdeen open canopy habitat despite greater total snail biomass in Yarra (Fig. 4).

Temporal dynamics likely moderate the impact of invasive snails on N cycling. This study was conducted during the dry season in the Caribbean, when stream discharge is relatively low and flooding events are typically infrequent. These conditions facilitate growth of primary producers that provide food resources for *T. granifera*. If snail biomass is associated with food quantity as our results suggest, areal snail excretion rates will be more pronounced during the dry season. In contrast, hydrologic variability due to frequent rain events in the wet season can diminish the impact of snail excretion through dilution and dislodgment of snails, reducing snail biomass and eroding spatial structure by washing individuals downstream. An analysis of shrimp N and P excretion [42] showed that variability in stream discharge constrained the influence of shrimp excretion rates on ambient NH4-N pools in Puerto Rican streams, providing evidence that temporal fluctuations in discharge can modulate the importance of excretion. Frequent scouring and streambed movement can also continually reset algal growth on benthic substrate, further diminishing the influence of riparian canopy during the wet season.

Understanding direct links between impacts of species introduction and habitat degradation is important for understanding the spatial dynamics and spread of invasive species as well as for prioritizing use of limited funding for control efforts. Eradication–if possible–is often arduous and costly, and careful examination is necessary to identify the most urgent cases that have potential for success. Habitat degradation can unintentionally create patches of favorable habitat that act as spatial resource subsides to invasive species at landscape scales. Our study suggests that populations of *T. granifera* can have a variable influence on nutrient cycling in tropical systems, dependant on light availability. Mitigation efforts focused on curtailing habitat degradation in adjacent riparian corridors may therefore ameliorate the magnitude of impacts. In this case, a management strategy involving restoration and/or protection of riparian buffer zones along stream corridors may decrease the biogeochemical impacts of an established non-native mollusk. Further investigation into interactive effects of human-mediated habitat degradation and introduced animals on nutrient cycles may uncover pragmatic management opportunities.

## Supporting Information

Figure S1 **Nutrient limitation in Ramdeen Stream.** Mean chlorophyll *a* on nutrient diffusing substrates after a two-week incubation in RAM. Asterisk indicates significantly greater chlorophyll *a* relative to controls using a randomized block ANOVA (*p*<0.001). Results revealed that algal accrual was co-limited by N and P availability, as substrates containing both N and P were the only treatment with significantly greater algal biomass than controls.
(TIF)

Figure S2 **Short-term NH_4_ addition in Ramdeen stream in 2008 and 2010.** Tracer NH_4_-N and conductivity are concentrations at plateau corrected for background concentrations. Distance from injection site indicates location downstream from site where solutes were added using a peristaltic pump.
(TIF)

Table S1 **Stoichiometry of** ***T. granifera*** **body tissue.** Mean (±1SE) body tissue C:N and C:P of *T. granifera* (shell removed) in open and closed canopy habitat. RAM  =  Ramdeen Stream, ARI  =  Aripo River.
(DOCX)

Supporting Information S1 **Nutrient limitation in Ramdeen Stream as measured using nutrient diffusing substrates.**
(DOCX)

Supporting Information S2 **Calculation of NH_4_-N uptake length, uptake velocity, and areal uptake rate in Ramdeen Stream.**
(DOCX)

Supporting Information S3 **Measurement of C, N, and P stoichiometry of** ***T. granifera*** **body tissue.**
(DOCX)

## References

[1] Vitousek PM, Mooney HA, Lubchenco J, Melillo JM (1997). Human domination of Earth’s ecosystems.. Science.

[2] Moulton LA, Pimm SL (1983). The introduced Hawaiian avifauna: biogeographic evidence for competition.. Am Nat.

[3] Vitousek PM (1990). Biological invasions and ecosystem processes: towards an integration of population biology and ecosystem studies.. Oikos.

[4] Baxter CV, Fausch KD, Murakami M, Chapman PL (2004). Fish invasion restructures stream and forest food webs by interrupting reciprocal prey subsidies.. Ecology.

[5] Vanni MJ (2002). Nutrient cycling by animals in freshwater ecosystems.. Annu Rev Ecol Syst.

[6] Hall Jr RO, Tank JL, Dybdahl MF (2003). Exotic snails dominate nitrogen and carbon cycling in a highly productive stream.. Front Ecol Environ.

[7] Arnott DL, Vanni MJ (1996). Nitrogen and phosphorus recycling by the zebra mussel (Dreissena polymorpha) in the western basis of Lake Erie.. Can J Fish Aquat Sci.

[8] McIntyre PB, Flecker AS, Vanni MJ, Hood JM, Taylor BW (2008). Fish distributions and nutrient cycling in streams: can fish create biogeochemical hotspots?. Ecology.

[9] Vanni MJ, Flecker AS, Hood JM, Headworth JL (2002). Stoichiometry of nutrient recycling by vertebrates in a tropical stream: linking species identity and ecosystem processes.. Ecol Lett.

[10] McIntyre PB, Jones LE, Flecker AS, Vanni MJ (2007). Fish extinctions alter nutrient recycling in tropical freshwaters.. Proc Natl Acad Sci USA.

[11] Small GE, Pringle CM, Pyron M, Duff JH (2011). Role of fish Astyanax aeneus (Characidae) as a keystone nutrient recycler in low-nutrient Neotropical streams.. Ecology.

[12] Carlsson NOL, Bronmark C, Hansson L (2004). Invading herbivory: the golden apple snail alters ecosystem functioning in Asian wetlands.. Ecology.

[13] Downing JA, McClain M, Twilley R, Melack JM, Elser J (1999). The impact of accelerating land use change on the N-cycle of tropical aquatic ecosystems: current conditions and projected changes.. Biogeochemistry.

[14] Elser JJ, Bracken MES, Cleland EE, Gruner DS, Harpole WS (2007). Global analysis of nitrogen and phosphorus limitation in freshwater, marine and terrestrial ecosystem.. Ecology Letters.

[15] Meyerson LA, Mooney HA (2007). Invasive alien species in an era of globalization.. Front Ecol Environ.

[16] Strayer DL (2010). Alien species in fresh waters: ecological effects, interactions with other stressors, and prospects for the future.. Freshwater Biol.

[17] Didham RK, Tylianakis JM, Gemmell NJ, Rand TA, Ewers RM (2007). Interactive effects of habitat modification and species invasion on native species decline.. Trends Ecol Evol.

[18] Likens GE, Bormann FH (1974). Linkages between terrestrial and aquatic ecosystems.. Bioscience.

[19] Nakano S, Miyasaka H, Kuhara N (1999). Terrestrial–aquatic linkages: riparian arthropod inputs alter trophic cascades in a stream food web.. Ecology.

[20] Flecker AS, Taylor BW, Bernhardt ES, Hood JM, Cornwell WK (2002). Interactions between herbivorous fishes and limiting nutrients in a tropical stream ecosystem.. Ecology.

[21] Pointier J, McCullough F (1989). Biological control of the snail hosts of Schistosoma mansoni in the Caribbean area using Thiara spp.. Acta Trop.

[22] Tank JL, Bernot MJ, Rosi-Marshall EJ, Hauer R, Lamberti GA (2006). Nitrogen limitation and uptake..

[23] Arar EJ, Collins GB (1997). Method 445.0. In vitro determination of chlorophyll *a* and pheophytin *a* in marine and freshwater algae by fluorescence.. U.S. Environmental Protection Agency, Cincinnati, OH.

[24] Wallace JB, Hutchens JJ, Grubaugh JW, Hauer R, Lamberti GA (2006). Transport and storage of FPOM..

[25] Taylor BW, Keep CF, Hall RO, Koch BJ, Tronstad LM (2007). Improving the fluorometric ammonium method: matrix effects, background fluorescence, and standard additions.. J N Am Benthol Soc.

[26] Snider SB (2007). Towards a movement ecology: modeling the behavioral response of invasive snails to resources and competition. PhD thesis.. North Carolina State University, Raleigh, NC.

[27] Hall Jr RO, Dybdahl MF, VanderLoop MC (2006). Extremely high secondary production of introduced snails in rivers.. Ecol Appl.

[28] Hedges LV, Gurevitch J, Curtis PS (1999). The meta-analysis of response ratios in experimental ecology.. Ecology.

[29] Newbold JD, Elwood JW, O’Neill RV, Van Winkle W (1981). Measuring nutrient spiraling in streams.. Can J Fish Aquat Sci.

[30] Mulholland PJ, Tank JL, Webster JR, Bowden WB, Dodds WK (2002). Can uptake length in streams be determined by nutrient addition experiments? Results from an interbiome comparison study.. J N Am Benthol Soc.

[31] Peterson BJ, Deegan L, Helfrich J, Hobbie JE, Hullar M (1993). Biological responses of a tundra river to fertilization.. Ecology.

[32] Wallace JB, Eggert SL, Meyer JL, Webster JR (1999). Effects of resource limitation on a detrital-based ecosystem.. Ecol Monogr.

[33] Hill WR, Smith JG, Stewart AJ (2010). Light, nutrients, and herbivore growth in oligotrophic streams.. Ecology.

[34] Rand TA, Tylianakis JM, Tscharntke T (2006). Spillover edge effects: the dispersal of agriculturally subsidized insect natural enemies into adjacent natural habitats.. Ecol Lett.

[35] Sterner RW, Elser JJ, Fee EJ, Guildford SJ, Chrzanowski TH (1997). The light:nutrient ratio in lakes: the balance of energy and materials affects ecosystem structure and process.. Am Nat.

[36] Frost PC, Elser JJ (2002). Effects of light and nutrients on the net accumulation and elemental composition of epilithon in boreal lakes.. Freshwater Biol.

[37] Hill WR, Fanta SE (2008). Phosphorus and light colimit periphyton growth at subsaturating irradiances.. Freshwater Biol.

[38] Liess A, Lange K, Schulz F, Piggott JJ, Matthaei CD (2009). Light, nutrients and grazing interact to determine diatom species richness via changes to productivity, nutrient state and grazer activity.. J Ecol.

[39] Sanches LF, Guariento RD, Caliman A, Bozelli RL, Esteves FA (2011). Effects of nutrients and light on periphytic biomass and nutrient stoichiometry in a tropical black-water aquatic ecosystem.. Hydrobiologia.

[40] Wootton JT, Parker MS, Power ME (1996). Effects of disturbance on river food webs.. Science.

[41] Hall Jr RO, Koch BJ, Marshall MC, Taylor BW, Trondstad LM, Hildrew AG, Edmonds-Brown R, Raffaelli D (2007). How body size mediates the role of animals in nutrient cycling in aquatic systems..

[42] Benstead JP, Cross WF, March JG, McDowell WH, Ramirez A (2010). Biotic and abiotic controls on the ecosystem significance of consumer excretion in two contrasting tropical streams.. Freshwater Biology.

