# Supplementary figures and images for: Impacts of an Invasive Snail (Tarebia granifera) on Nutrient Cycling in Tropical Streams: The Role of Riparian Deforestation in Trinidad, West Indies
Source: PLoS One. 2012 Jun 25;7(6):e38806. doi: 10.1371/journal.pone.0038806 (PMC3382606; doi:10.1371/journal.pone.0038806)

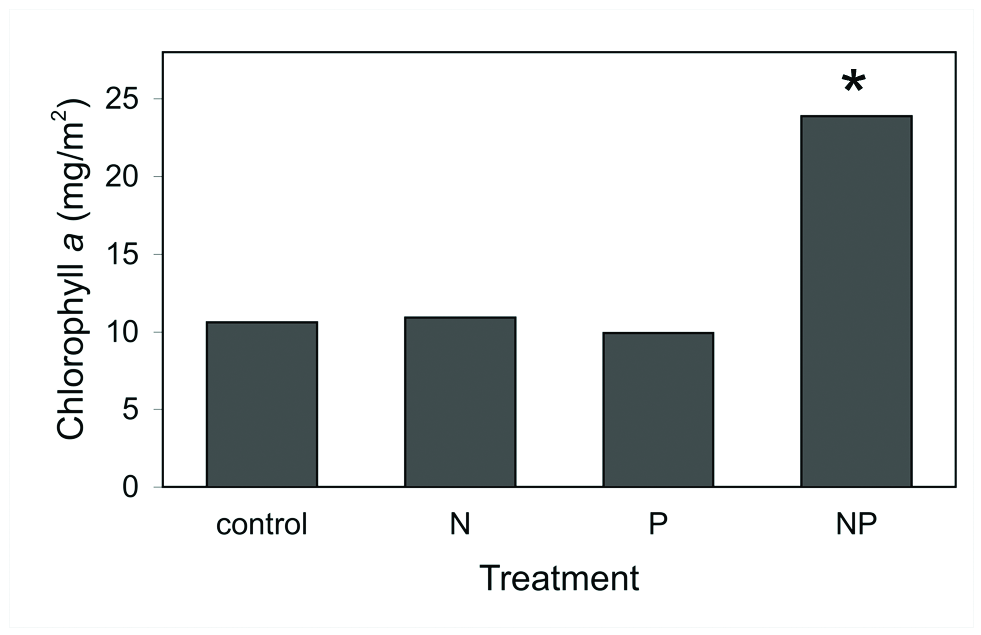

Supplement: Figure S1 — Nutrient limitation in Ramdeen Stream. Mean chlorophyll a on nutrient diffusing substrates after a two-week incubation in RAM. Asterisk indicates significantly greater chlorophyll a relative to controls using a randomized block ANOVA (p<0.001). Results revealed that algal accrual was co-limited by N and P availability, as substrates containing both N and P were the only treatment with significantly greater algal biomass than controls. (TIF) [file pone.0038806.s001.tif]

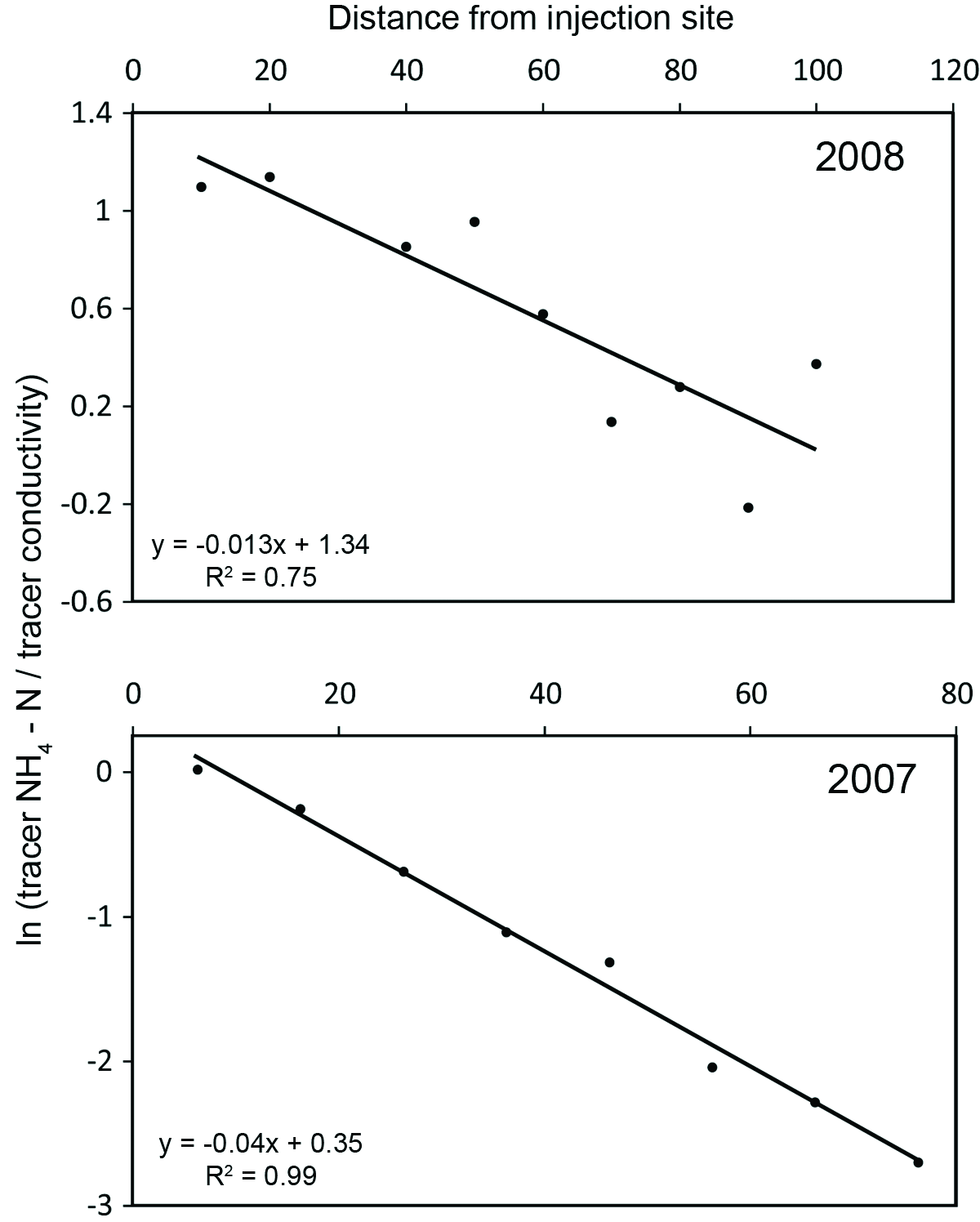

Supplement: Figure S2 — Short-term NH4 addition in Ramdeen stream in 2008 and 2010. Tracer NH4-N and conductivity are concentrations at plateau corrected for background concentrations. Distance from injection site indicates location downstream from site where solutes were added using a peristaltic pump. (TIF) [file pone.0038806.s002.tif]
