# Supplementary material for: Impacts of an Invasive Snail (Tarebia granifera) on Nutrient Cycling in Tropical Streams: The Role of Riparian Deforestation in Trinidad, West Indies
Source: PLoS One. 2012 Jun 25;7(6):e38806. doi: 10.1371/journal.pone.0038806 (PMC3382606; doi:10.1371/journal.pone.0038806)
Supplement: Table S1 — Stoichiometry of T. granifera body tissue. Mean (±1SE) body tissue C:N and C:P of T. granifera (shell removed) in open and closed canopy habitat. RAM = Ramdeen Stream, ARI = Aripo River. (DOCX) [file pone.0038806.s003.docx]

|  | RAM | | ARI | |
| --- | --- | --- | --- | --- |
|  | C:N | C:P | C:N | C:P |
| Closed canopy | 5.3 + 0.3 | 114 + 9 | 6.0 + 0.3 | 93 + 3 |
| Open canopy | 5.4 + 0.3 | 116 + 10 | 5.5 + 0.2 | 104 + 5 |
