# Supplementary material for: Impacts of an Invasive Snail (Tarebia granifera) on Nutrient Cycling in Tropical Streams: The Role of Riparian Deforestation in Trinidad, West Indies
Source: PLoS One. 2012 Jun 25;7(6):e38806. doi: 10.1371/journal.pone.0038806 (PMC3382606; doi:10.1371/journal.pone.0038806)
Supplement: Supporting Information S1 — Nutrient limitation in Ramdeen Stream as measured using nutrient diffusing substrates. (DOCX) [file pone.0038806.s004.docx]

**Supporting information S1.**

Nutrient diffusing substrates (NDS) were constructed following methods in Tank et al. (2006) and placed in RAM in 2007. NDS were constructed using 30 ml plastic canisters filled with agar gel amended with nutrient salts. The NDS analysis consisted of four nutrient treatments: a control (no nutrients added), nitrogen (N) added, phosphorus (P) added, and both N and P added. N was added in the form of NH_4_NO_3_ and P was added in the form of KH_2_PO_4_ to reach a concentration of 0.5 M for each compound. The agar gel regulated diffusion of nutrients through a fitted glass disc (glass crucible covers, Leco Corporation, St. Joeseph, Michigan, USA) that acted as a growth surface. The fritted glass disc was held in place by a tight fitting cap with a 2.2 cm diameter circular hole to expose the growth surface to the water column. Substrates were attached in a random sequence to plastic bars (two bars of 12 NDS each, six total replicates for each treatment) and bars were secured to the stream bottom using metal stakes. Substrates were collected after an in-stream incubation period of two weeks and algal accrual was quantified as concentration of chlorophyll *a*. Glass discs were removed and extracted in a 90% buffered ethanol solution for 24 h and concentration of chlorophyll *a* in the solution was quantified using a Turner Designs Aquafluor fluorometer (Sunnyvale, CA, USA) following methods in Arar and Collins (1997).

For statistical analyses of nutrient limitation, we used a randomized block ANOVA with nutrient treatment as a fixed effect and bar number to which canisters were attached as a random effect followed by Tukey’s HSD multiple comparison tests (SAS Institute, 2009). Results revealed that algal accrual was co-limited by N and P availability, as substrates containing both N and P were the only treatment with significantly greater algal biomass than controls (*P* < 0.001, Fig. S1).

Arar, E. J. and G. B. Collins. 1997. Method 445.0. In vitro determination of chlorophyll *a* and pheophytin *a* in marine and freshwater algae by fluorescence. U.S. Environmental Protection Agency, Cincinnati, OH.
