# Supplementary material for: Impacts of an Invasive Snail (Tarebia granifera) on Nutrient Cycling in Tropical Streams: The Role of Riparian Deforestation in Trinidad, West Indies
Source: PLoS One. 2012 Jun 25;7(6):e38806. doi: 10.1371/journal.pone.0038806 (PMC3382606; doi:10.1371/journal.pone.0038806)
Supplement: Supporting Information S2 — Calculation of NH4-N uptake length, uptake velocity, and areal uptake rate in Ramdeen Stream. (DOCX) [file pone.0038806.s005.docx]

**Supporting information S2**

Uptake length, or average distance a NH_4_ molecule travels downstream before it is taken up by biota, was calculated using the exponential decay model ln *N_x_* = ln *N_0_* – *kx*, where *N_0_* and *N_x_* are background-corrected NH_4_ concentrations at the addition site and *x* meters downstream from the addition site, *k* is the exponential decay rate, and uptake length is 1/*k* (Newbold et al. 1981; *p* < 0.01, Fig. S2). Uptake length measurements vary with stream discharge as greater discharge will carry nutrient molecules further before they make contact with the streambed and are incorporated by biota. To control for variation in discharge we calculated NH_4_ uptake velocity, or NH_4_ demand relative to NH_4_ concentration in streamwater, as V_f_ (m/min) = *Qk*/*w* where *Q* is discharge (m^3^/min), *k* is the inverse of uptake length, and *w* is mean stream width. We used the uptake velocity estimate to calculate areal uptake rate as *U* (mgN/m^2^/h) = V_f_N_b_ * 60min/h, where N_b_ is mean background NH_4_ concentration (*n* = 10) before solutes were added to the stream (Newbold et al. 1981).

Newbold, J. D., J. W. Elwood, R. V. O'Neill, and V. W. W. 1981. Measuring nutrient spiraling in streams. Canadian Journal of Fisheries and Aquatic Sciences 38:860-863.
