# Supplementary material for: Impacts of an Invasive Snail (Tarebia granifera) on Nutrient Cycling in Tropical Streams: The Role of Riparian Deforestation in Trinidad, West Indies
Source: PLoS One. 2012 Jun 25;7(6):e38806. doi: 10.1371/journal.pone.0038806 (PMC3382606; doi:10.1371/journal.pone.0038806)
Supplement: Supporting Information S3 — Measurement of C, N, and P stoichiometry of T. granifera body tissue. (DOCX) [file pone.0038806.s006.docx]

**Supporting information S3**

For elemental composition analysis of snail body tissue, shells were removed and body tissue dried to a constant weight, ground to a fine powder, and homogenized using a mortar and pestle. Subsamples were weighed on a microbalance (Mettler Toledo MX5) to the nearest μg and analyzed for C and N content using a CHNS elemental analyzer (Elementar Vario EL III). For P content, subsamples were weighed into acid-washed Pyrex tubes, ashed at 500 °C, digested in 1N HCl, and analyzed on a Shimadzu UV 1240 spectrophotometer (molybdate blue method, Murphy and Riley 1962). Ground citrus leaves and spinach (US National Institute of Standards and Technology, US Department of Congress) were used as standards for P analysis and were analyzed in each set of samples. Percent recovery of P from standards was typically 97-100%. Elemental ratios are molar.
